# Supplementary material for: Factors associated with barriers to healthcare access among ever-married women of reproductive age in Bangladesh: Analysis from the 2017–2018 Bangladesh Demographic and Health Survey
Source: PLoS One. 2024 Jan 5;19(1):e0289324. doi: 10.1371/journal.pone.0289324 (PMC10769052; doi:10.1371/journal.pone.0289324)
Supplement: S1 Table — (DOCX) [file pone.0289324.s001.docx]

**Supplementary table 1:** Background characteristics of barriers to healthcare access among women in Bangladesh and Crude odds ratio (n = 20,127)

| characteristics | Permission | Money | Distance | Alone | At least one barrier |
| --- | --- | --- | --- | --- | --- |
|  | COR [95%CI] | COR [95%CI] | COR [95%CI] | COR [95%CI] | COR [95%CI] |
| **Age** |  |  |  |  |  |
| 15-19 | 1.23 [1.04-1.46]* | 0.54 [0.48-0.61]*** | 0.90 [0.80-1.01] | 1.31 [1.16-1.47]*** | 0.88 [0.77-1.00] |
| 20-24 | 0.88 [0.75-1.03] | 0.57 [0.51-0.64]*** | 0.86 [0.77-0.96]** | 0.93 [0.84-1.04] | 0.67 [0.60-0.75]*** |
| 25-29 | 0.90 [0.77-1.05] | 0.70 [0.63-0.78]*** | 0.90 [0.81-1.00]* | 0.88 [0.79-0.98]* | 0.76 [0.68-0.85]*** |
| 30-34 | 0.78 [0.67-0.92]** | 0.82 [0.74-0.91]*** | 0.98 [0.88-1.09] | 0.85 [0.77-0.95]** | 0.80 [0.71-0.89]*** |
| 35-39 | 0.87 [0.74-1.03] | 0.91 [0.82-1.02] | 0.98 [0.88-1.10] | 0.86 [0.77-0.97]* | 0.86 [0.77-0.97] |
| 40-44 | 0.75 [0.62-0.89]** | 0.92 [0.82-1.03] | 0.94 [0.84-1.06] | 0.93 [0.83-1.05] | 0.89 [0.79-1.01] |
| 45-49 | Ref | Ref | Ref | Ref | Ref |
| **Marital status** |  |  |  |  |  |
| Married | - | Ref |  |  | Ref |
| Widowed | - | 1.87 [1.59-2.20]*** | - | - | 1.28 [1.07-1.53]** |
| Divorced | - | 1.79 [1.43-2.26]*** | - | - | 1.10 [0.86-1.40] |
| No longer living together /separated | - | 2.15 [1.64-2.83]*** | - | - | 1.36 [1.01-1.84]* |
| **Educational level** |  |  |  |  |  |
| No education | Ref | Ref | Ref | Ref | Ref |
| Primary | 0.90 [0.80-1.02] | 0.70 [0.64-0.76]*** | 0.83 [0.76-0.90]*** | 0.94 [0.87-1.02] | 0.80 [0.73-0.88]*** |
| Secondary | 0.82 [0.73-0.92]** | 0.37 [0.34-0.41]*** | 0.65 [0.60-0.71]*** | 0.83 [0.77-0.90]*** | 0.52 [0.48-0.57]*** |
| Higher | 0.45 [0.37-0.54]*** | 0.15 [0.13-0.17]*** | 0.40 [0.35-0.44]*** | 0.49 [0.44-0.55]*** | 0.25 [0.22-0.28]*** |
| **Occupation** |  |  |  |  |  |
| Not working | Ref | Ref | Ref | Ref | Ref |
| Professional /technical /managerial | 0.37 [0.24-0.58]*** | 0.25 [0.18-0.34]*** | 0.47 [0.37-0.60]*** | 0.36 [0.28-0.46]*** | 0.34 [0.28-0.43]*** |
| Sales | 0.88 [0.62-1.24] | 1.26 [1.00-1.58]* | 0.96 [0.76-1.21] | 0.69 [0.54-0.87]** | 0.96 [0.76-1.21] |
| Agricultural | 0.79 [0.72-0.87]*** | 1.77 [1.66-1.89]*** | 1.44 [1.36-1.54]*** | 1.23 [1.15-1.31]*** | 1.73 [1.62-1.86]*** |
| Household and domestic | 0.81 [0.58-1.13] | 3.75 [2.99-4.70]*** | 1.17 [0.95-1.45] | 0.93 [0.76-1.15] | 1.72 [1.36-2.19]*** |
| Services | 0.68 [0.54-0.86]** | 1.51 [1.31-1.73]*** | 0.87 [0.75-1.00] | 0.78 [0.67-0.89]*** | 1.01 [0.88-1.17] |
| Manual | 0.58 [0.48-0.70]*** | 1.41 [1.26-1.56]*** | 0.93 [0.84-1.04] | 0.85 [0.76-0.95]** | 1.15 [1.03-1.29]* |
| **Religion** |  |  |  |  |  |
| Islam | - | Ref | Ref | Ref | Ref |
| Hinduism | - | 1.18 [1.07-1.30]** | 1.21 [1.10-1.34]*** | 1.19 [1.07-1.31]** | 1.22 [1.10-1.36]*** |
| Other (Buddhism & Christianity) | - | 2.16 [1.55-3.01]*** | 1.71 [1.24-2.36]** | 1.35 [0.98-1.87] | 2.00 [1.34-2.99]** |
| **Covered by health insurance** |  |  |  |  |  |
| No | - | - | - | - | - |
| Yes | - | - | - | - | - |
| **Frequency of listening to radio** |  |  |  |  |  |
| Not at all | - | Ref | Ref | Ref | Ref |
| Less than once a week | - | 0.45 [0.37-0.54]*** | 0.67 [0.56-0.80]*** | 0.83 [0.70-0.98]* | 0.59 [0.49-0.69]*** |
| At least once a week | - | 0.45 [0.36-0.56]*** | 0.59 [0.48-0.73]*** | 0.72 [0.58-0.87]** | 0.57 [0.47-0.69]*** |
| **Frequency of reading newspaper or magazine** |  |  |  |  |  |
| Not at all | Ref | Ref | Ref | Ref | Ref |
| Less than once a week | 0.62 [0.50-0.77]*** | 0.32 [0.28-0.37]*** | 0.53 [0.46-0.60]*** | 0.58 [0.51-0.65]*** | 0.41 [0.36-0.46]*** |
| At least once a week | 0.39 [0.28-0.56]*** | 0.22 [0.18-0.27]*** | 0.41 [0.34-0.50]*** | 0.36 [0.30-0.43]*** | 0.26 [0.22-0.30]*** |
| **Frequency of watching television** |  |  |  |  |  |
| Not at all | Ref | Ref | Ref | Ref | Ref |
| Less than once a week | 1.22 [1.06-1.41]** | 0.84 [0.76-0.93]** | 0.75 [0.68-0.83]*** | 0.75 [0.68-0.83]*** | 0.84 [0.75-0.94]** |
| At least once a week | 0.78 [0.71-0.86]*** | 0.48 [0.45-0.51]*** | 0.57 [0.53-0.60]*** | 0.66 [0.62-0.70]*** | 0.52 [0.49-0.55]*** |
| **Owns a mobile telephone** |  |  |  |  |  |
| No | Ref | Ref | Ref | Ref | Ref |
| Yes | 0.72 [0.66-0.79]*** | 0.53 [0.50-0.56]*** | 0.73 [0.69-0.77]*** | 0.71 [0.67-0.75]*** | 0.56 [0.52-0.59]*** |
| **Sex of household head** |  |  |  |  |  |
| Male | - | - | - | Ref | - |
| Female | - | - | - | 0.89 [0.83-0.97]** | - |
| **Wealth index** |  |  |  |  |  |
| Poorest | Ref | Ref | Ref | Ref | Ref |
| Poorer | 0.86 [0.75-0.98]* | 0.63 [0.58-0.69]*** | 0.75 [0.69-0.82]*** | 0.89 [0.81-0.97]** | 0.76 [0.69-0.85]*** |
| Middle | 0.94 [0.82-1.07] | 0.40 [0.36-0.44]*** | 0.61 [0.56-0.67]*** | 0.81 [0.74-0.89]*** | 0.53 [0.48-0.59]*** |
| Richer | 0.78 [0.68-0.89]*** | 0.28 [0.26-0.31]*** | 0.48 [0.44-0.53]*** | 0.73 [0.67-0.80]*** | 0.42 [0.38-0.47]*** |
| Richest | 0.51 [0.44-0.59]*** | 0.17 [0.16-0.19]*** | 0.32 [0.29-0.35]*** | 0.48 [0.44-0.52]*** | 0.25 [0.22-0.27]*** |
| **Residence** |  |  |  |  |  |
| Urban | Ref | Ref | Ref | Ref | Ref |
| Rural | 1.54 [1.39-1.71]*** | 1.42 [1.33-1.51]*** | 1.82 [1.71-1.94]*** | 1.55 [1.46-1.65]*** | 1.73 [1.63-1.85]*** |
| **Region** |  |  |  |  |  |
| Dhaka | Ref | Ref | Ref | Ref | Ref |
| Barisal | 1.39 [1.10-1.75]** | 0.77 [0.67-0.89]*** | 0.64 [0.56-0.74]*** | 0.76 [0.66-0.88]*** | 0.66 [0.56-0.77]*** |
| Chittagong | 1.45 [1.16-1.82]** | 0.84 [0.73-0.96]* | 0.71 [0.62-0.81]*** | 0.81 [0.71-0.93]** | 0.75 [0.65-0.87]*** |
| Khulna | 1.11 [0.87-1.43] | 1.01 [0.87-1.17] | 0.83 [0.72-0.97]* | 1.01 [0.87-1.17] | 1.03 [0.88-1.22] |
| Mymensingh | 0.80 [0.60-1.07] | 1.21 [1.03-1.42]* | 0.75 [0.63-0.88]*** | 0.84 [0.71-0.98]* | 0.86 [0.72-1.03] |
| Rajshahi | 1.11 [0.87-1.42] | 0.73 [0.63-0.84]*** | 0.58 [0.50-0.67]*** | 0.70 [0.61-0.81]*** | 0.70 [0.60-0.82]*** |
| Rangpur | 1.66 [1.31-2.11]*** | 1.28 [1.10-1.14]** | 0.87 [0.75-1.01] | 0.93 [0.80-1.08] | 1.14 [0.96-1.34] |
| Sylhet | 1.36 [1.03-1.79]* | 0.96 [0.81-1.14] | 0.88 [0.74-1.04] | 0.95 [0.80-1.13] | 0.86 [0.71-1.03] |
| Not dejure resident | 1.37 [1.06-1.77]* | 0.60 [0.51-0.71]*** | 0.60 [0.52-0.71]*** | 0.79 [0.67-0.92]** | 0.56 [0.47-0.66]*** |

*p<0.05, **<0.01, ***<0.001

COR: crude odds ratio, ref: reference
