# Supplementary material for: Factors associated with barriers to healthcare access among ever-married women of reproductive age in Bangladesh: Analysis from the 2017–2018 Bangladesh Demographic and Health Survey
Source: PLoS One. 2024 Jan 5;19(1):e0289324. doi: 10.1371/journal.pone.0289324 (PMC10769052; doi:10.1371/journal.pone.0289324)
Supplement: Attachment — Submitted filename: Comment.docx [file pone.0289324.s002.docx]

Title: Factors associated with barriers to healthcare access among ever-married women of reproductive age in Bangladesh: analysis from the 2017-2018 Bangladesh Demographic and Health Survey

Authors: Hitomi Hinata et al.

Objective: to examine the association between social determinants and barriers to accessing healthcare among ever-married women aged 15-49 in Bangladesh.

Overall impression: The article is interesting, and I think it will contribute to policy-making. I have some observations. I hope it will add value to the paper if the authors considered it in revision. I would recommend publishing it after major revision.

Comments:

1. Abstract: in background: Please specify what remarkable changes have been made by Bangladesh if possible in numeric value.
2. Abstract: Methods: It would be better to provide the independent variables and how did you analyze them? Also, provide the statistical software that you used for analysing the data. Did you manipulate the variables and data? If yes provide the information?
3. Abstract: Result: Present the result of bivariate analysis also.
4. Abstract: Conclusions: Pinpoint how the policy-makers can utilize your result.
5. Data Availability: I would recommend writing as 'Data will be freely available after the reasonable request from the DHS program'. Since it is not possible to access the data without a request.
6. Introduction: Can you present the findings of previous BDHSs? Were there any changes and which variables are remarkably changed compared to previous one? What are the possible factors for that?
7. Materials and methods: More elaboration is needed here. Follow STROBE guidelines. I am confusing the sentence: 'In the second stage, 20,2500 households were selected for data collection,….' Please revisit it.
8. Statistical analysis: Citation required here at: ..' STATA/MP version 17.0 was used for analysis.' How did you assess the variables in multivariate analysis? Did you assess the multi-collinearity issue? What is the basis of assessing the variables?
9. Statistical analysis: In the multivariate analysis, there is no consistency in assuming reference value? For example, last attribute/category in age group, while first category/attributes for others? What is the cause of that? Sometimes, it will be interesting if you make the middle attribute as reference category such as in wealth quintile.
10. Result: Present the impression of the table rather than an explanation of the table exactly. What is the basis of 5-year age category?
11. Result: … 5.7% of respondents were widowed, divorced..' Do not start the sentence with number. You may write as 'almost six (5.7) percent of the total respondents…'

In table 3: Wealth Index: The reference category in the middle would be interesting to compare with the poorest and the richest.

12. Result: In table 3: Residence: Rural woman OR = 0.77. It is interesting. Are the rural women rich in Bangladesh? ….The women who lived in rural areas had less likely to have financial barrier compared to those women who lived in urban areas...Am I right? Please present the possible causes for that.
13. Result in table 3: It is not necessary to indicate ** for significant value. The value of 95%CI shows whether it is significant or not. Present the model summary of each dependent variables. It shows the predictive capacity of the table. If possible provide value of AUR(Area Under Curve).
14. Discussion: Present your discussion theme wise i.e. Autonomy, Money, Proximity, Alone etc. Add these themes as sub heading of the discussion section.
15. Discussion: ..'Our study identified that the proportion of the barriers to access to healthcare in Bangladesh…' Present the exact value.
16. Conclusion: Please be specific while suggesting to the policy-makers. How your findings can be translated into policy-making?
17. Ethical Issue: I think, ethical approval was taken by organization while conducting BDHS.
18. Ethical approval and consent to participate: I would recommend to write as '…. The survey protocol was approved by institutional review boards (IRBs) at ICF and the Bangladesh Medical Research Council (BMRC). Both IRBs and BMRC approved the protocols before the commencement of data collection activities.. Consent was taken before administered…. '
19. Data availability: Revise your sentence. It is not possible to access the data without login and proposal. There is no data accessible through the link.
20. Others: Merge the citation as required. Provide links or DOI as possible and applicable so that readers could visit the original sources easily. Revisit the paper for grammar and sentence structure. Somewhere, there is no consistency within and among the sentences and paragraphs.
